# Resilience of the primary healthcare system: perspectives of German stakeholders at primary care interfaces during the second wave of the COVID-19 pandemic

**DOI:** 10.3389/fmed.2024.1322765

**Published:** 2024-04-24

**Authors:** Nicola Litke, Sophia Weber, Amanda Breckner, Catharina Roth, Frank Peters-Klimm, Michel Wensing, Sandra Stengel

**Affiliations:** Department of General Practice and Health Services Research, Heidelberg University Hospital, Faculty of Medicine, Heidelberg University, Heidelberg, Germany

**Keywords:** COVID-19 pandemic, primary care, primary health care, resilience, interface, coordination

## Abstract

**Introduction:**

Worldwide, the primary care sector played a key role in coping with the challenges of the SARS-CoV-2 pandemic.

**Methods:**

The aim of this study was to explore the resilience of the German primary healthcare system during the second wave of the pandemic from the perspective of identified interface stakeholders, i.e., representatives of hospitals, district offices, and medical associations. Qualitative data from interviews and focus groups were analyzed according to a resilience framework.

**Results:**

The main findings include a gap in knowledge transfer, unstructured allocation of responsibilities, and a resulting unregulated flow of patients. Social brokers supported care coordination and knowledge transfer. The response to the capacity to cope with uncertainties was slow and chaotic at the system level and heterogeneous at the individual level. Building on previous relationships fostered functioning communication, while competition in primary care was identified as a barrier to resilience.

**Conclusion:**

Implications for further research and for strengthening the resilience of primary care can be derived based on this study.

## 1 Introduction

Due to its rapid spread worldwide, the outbreak of the coronavirus disease 2019 (COVID-19) was officially declared as a pandemic by the World Health Organization on 11 March 2020 (1), which was perceived as a crisis by German primary care (2). Worldwide, the primary care sector played a key role in coping with the challenges of pandemic medical care and the health of the population (3–7). Successful coordination between sectors and institutions is required to ensure appropriate patient care in both usual care and pandemic conditions, resulting in multiple interfaces (5, 8–11). However, an analysis of the coordination and cooperation at these interfaces was missing (12).

Pandemic plans provide support at several levels (8, 9, 13, 14). In line with Germany's federal structure, each state has its own crisis plan, with Baden-Wuerttemberg being the third largest state. According to its pandemic plan, medical care should be provided predominantly and for as long as possible on an outpatient basis to relieve the burden on hospitals, so that resources in hospitals are sufficient for seriously ill patients. Nursing home residents with COVID-19 should be treated in their nursing homes as long as their health condition permits (8–10). According to the plan, several players were involved in the management of a pandemic: (i) the outpatient sector; (ii) the inpatient sector; (iii) the health authorities as part of the district offices with responsibility for contact tracing, assessment of the regional epidemiological situation, and advice and health information; (iv) the medical association with responsibility for advice and information (10); and (v) the Association of Statutory Health Insurance Physicians (ASHIP) with obligation to ensure provision of care (§§ 69 ff. SGB V). However, the specification of a plan is not necessarily followed by its implementation in practice.

For a healthcare system to be able to provide an adequate response to a crisis, it needs resilience (11, 15–18). “Resilience” is defined as the “capacity of a health system to absorb, adapt and transform when exposed to a shock such as a pandemic, natural disaster or armed conflict and still retain the same control over its structure and functions” (16). According to the authors, the resilience of a healthcare system is driven by the four main dimensions of knowledge, coping with uncertainties, interdependence, and legitimacy of institutions and norms (16), which implies the importance of changing care organizations and processes (7). Saulnier et al. (19) suggest including different stakeholders in a shared bottom-up process to describe the resilience of healthcare systems. Thus, the perspectives of pandemic interface stakeholders identified by primary care providers in Germany (16) complement the previously reported response. This is crucial to analyze the resilience of the primary care system during the early COVID-19 pandemic (20). Haldane et al. (21) described several limitations of the health system's resilience within the COVID-19 pandemic, but primarily on a health system level. Other studies analyzing resilience also focus mainly on system-level (15, 22) or individual resilience (23–25) rather than organizational resilience on the level of service delivery. Furthermore, proper applications of resilience theory in empirical practice are still lacking (15).

The aim of this explorative study was therefore to identify factors influencing the resilience of the German primary healthcare system from the perspective of identified interface stakeholders during the second wave of the SARS-CoV-2 pandemic.

## 2 Method

This qualitative study was part of the PrimaryCovCare project in the joint project “Lessons learned—Studie MWK COVID-19” with the university Institutes of General Practice Freiburg, Heidelberg, Tuebingen (lead), and Ulm. Qualitative, semi-structured online focus groups and interviews were carried out in an exploratory observational study with primary care interface stakeholders.

The study was funded by the Baden-Wuerttemberg Ministry for Science, Research, and the Arts as part of the larger project, Lessons learned—Studie MWK COVID-19, undertaken by the Departments of General Practice at the Medical Faculties of Baden-Wuerttemberg. The study is registered in the German Clinical Trial Register (DRKS00022224).

The COREQ criteria were used as a checklist for the report of this study (26) (Data sheet 1).

### 2.1 Study sample

Study participants were recruited by two researchers from the project team between September 2020 and January 2021 [the second wave of the COVID-19 pandemic (27)]. Initially, a targeted recruitment strategy was used by contacting the management of the institutions in Baden-Wuerttemberg by mail and asking for referrals (ASHIP, health authorities, district offices, randomly selected clinics, and nursing homes) to represent as much variation as possible from different regions. Due to recruitment difficulties in the middle of the stressful second wave of the pandemic, a pragmatic sampling strategy using the personal contacts of the study team was adopted. Primary care interface stakeholders included representatives of clinics, district offices and their health authorities (regional), medical associations, nursing homes, and the ASHIP of Baden-Wuerttemberg. All participants had to be at least 18 years old and able to give informed consent. If the feedback was positive, study information was provided by phone, mail, and email, and written consent was obtained from each participant.

### 2.2 Data collection and measures

Data collection was conducted by SW and SS between November 2020 and February 2021 [the second wave and early third wave of the COVID-19 pandemic (27)]. SW is a former female master's student with a background in health services research, and SS is a female post-doctoral general practitioner, a researcher in the field of general practice in the context of COVID-19, and experienced in qualitative research. Focus groups were conducted as the primary data source, but due to recruitment problems during the pandemic and the resulting high clinic workload, two individual telephone interviews were conducted. The online focus groups were conducted using Webex^®^ and the university's internal videoconferencing platform heiCONF^®^. Interviews and focus groups were audio-recorded, supplemented using protocol, and subsequently transcribed verbatim. No interview or focus group was repeated or canceled, and no transcript was returned to the participants for correction. All participants were also asked to complete a socio-demographic form.

A semi-structured interview guide was developed by an interprofessional team based on the results of previous literature research and experience from a previous project (20, 28). The interview guide included open questions on four main topics: (i) perception of cooperation with primary care physicians at the beginning of the pandemic; (ii) examples of best practices in collaboration with primary care physicians; (iii) ideas for improving cooperation with primary care physicians; and (iv) desire for continued development during the pandemic. In advance, a pilot focus group was conducted with two participants, and organizational processes were subsequently adjusted.

### 2.3 Data analysis

First, SW analyzed the data for thematic orientation. In a second step, NL and SS identified the resilience framework of Blanchet et al. (16) as appropriate for the data and research question and deductively coded the data following Braun and Clarke's Thematic Analysis (29). NL is a female researcher and doctoral candidate with a background in health services research, interprofessional healthcare, and speech and language therapy with experience in qualitative research.

NL and SS familiarized themselves with the data material. To start the coding phase, one of the focus groups was initially coded by NL and SS separately. Intercoder reliability was then detected, and a consensus meeting was held to thoroughly discuss differences in coding and interpretation until intercoder reliability reached 90%. This process was repeated for two more focus groups until high intercoder reliability was achieved immediately after coding. In addition, objective experts in qualitative research were included in the consensus meetings, and specific codes and interpretations of the framework were also discussed. After this initial coding phase, the remaining transcripts were primarily analyzed by either NL or SS. Then, in a second analysis step, each transcript was proofread and recoded by the other researcher who did not conduct the primary analysis.

Based on the resilience framework of Blanchet et al. (16), four main dimensions of managing resilience were primarily used for coding: (i) the capacity to combine and integrate different forms of knowledge, (ii) the ability to anticipate and cope with uncertainties and surprises, (iii) the capacity to manage interdependence: to engage effectively with and handle multiple- and cross-scale dynamics and feedback, and (iv) the capacity to build or develop legitimate institutions that are socially accepted and contextually adapted. Inductive subthemes were supplemented.

Within these four domains, generated codes were also analyzed regarding the capacity to absorb, adapt, and transform, as this represents three levels of resilience (16). Aspects were coded at first appearance, with no repetitive coding.

## 3 Results

A total of 37 participants were interviewed in seven focus groups with an average duration of 77 min (min. 58, max. 91) and two individual telephone interviews lasting 29 and 31 min, respectively. Table 1 presents the detailed characteristics of the participants.

**Table 1:** Sociodemographic characteristics.

	***n* (%)**
Total	37 (100%)
**Gender**	
Female	17 (45.9%)
Male	20 (54.0%)
**Region**	
North Baden	21 (56.8%)
North Wuerttemberg	6 (16.2%)
South Baden	4 (10.8%)
South Wuerttemberg	4 (10.8%)
No information	2 (5.4%)
**Professional background**	
(Local) medical association	8^*^ (21.6%)
Clinics	11 (29.7%)
Nursing homes	6 (16.2%)
Association of Statutory Health Insurance Physicians	5^**^ (13.5%)
District offices/health authorities	7 (18.9%)
**Age in years**	
Mean (standard deviation)	48.6 (11.6)
Minimum	23
Maximum	70

^*^Six primary care physicians, two specialists.

^**^Two primary care physicians; three institution employees.

**Figure 1:**
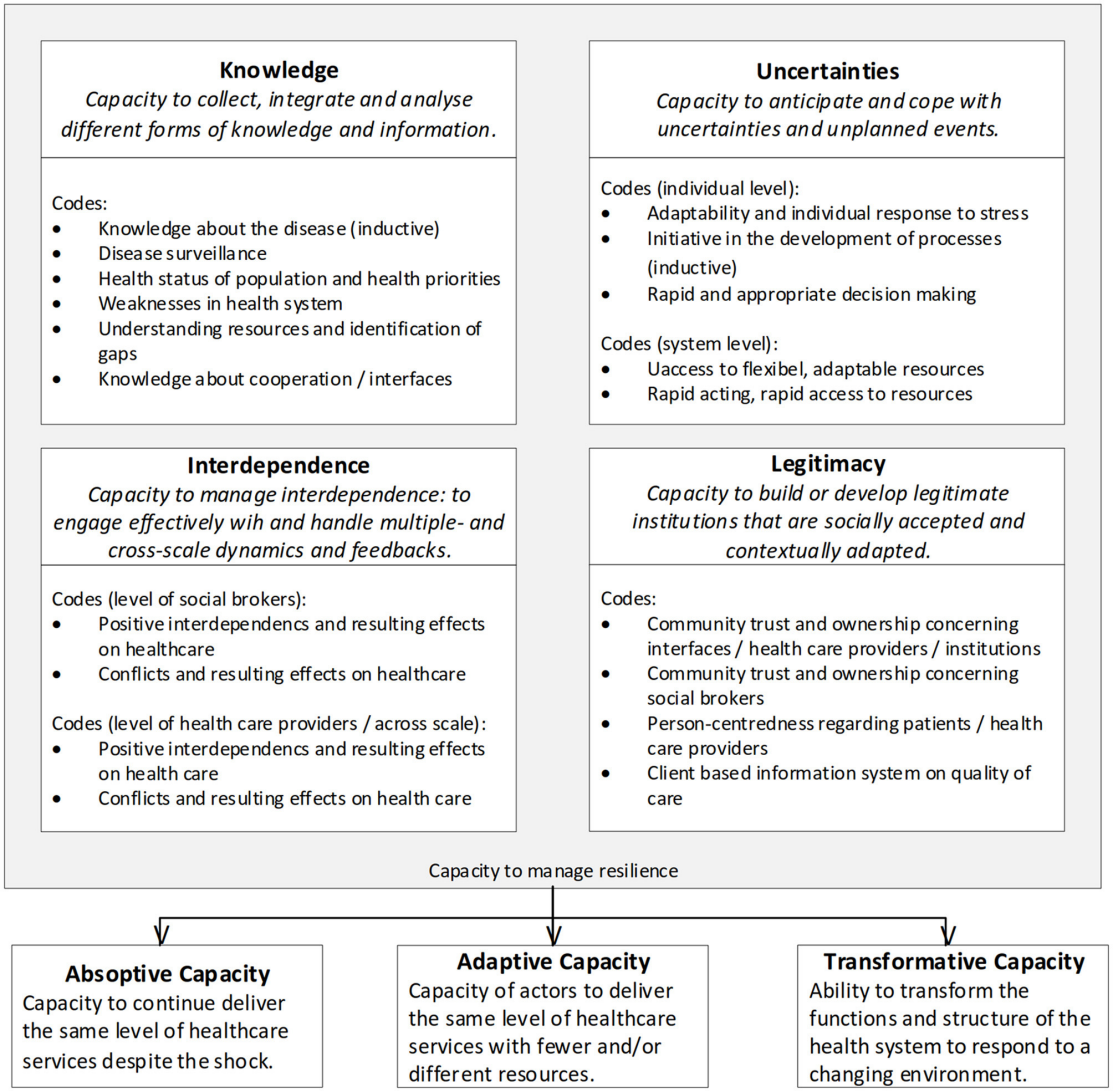
Overview of the detected codes, arranged according to the resilience framework by Blanchet et al. (16).

Statements resulted in all four main dimensions of the resilience framework. Figure 1 provides an overview of all dimensions and levels of the resilience framework and detected codes.

Most codes (491) were assigned to the dimension of knowledge. Describing interdependence resulted in 392 codes and the ability to cope with uncertainties in 312 codes. The least codes were assigned to the dimension of legitimacy (133 codes). Inductively, codes referring to the phase of recovery, like lessons learned or reflections of which strategies will be continued and which were not successful, occurred as well (149 codes). This aspect was not included in this manuscript.

An overview of the results is accessible in Data sheet 2.

### 3.1 The capacity to combine and integrate different forms of knowledge

Disease surveillance only plays a minor role in these data. Whenever it was mentioned, participants described times when “*none of us knew about actual case numbers*” (medical association 4, female, general practitioner).^1^ Information sharing was perceived as difficult due to problems with data security and a lack of digitization. This was related to both data retrieval and the reporting of diagnoses. There was also a lack of knowledge about how to deal with the virus and how to manage it at the system level. The information provided, for example, by the ASHIP, was described as heterogeneous and constantly changing.

From the participants' point of view, there was a lack of overview of institutions and practitioners and their responsibilities. A perceived lack of responsibility was described at all levels. It was not clearly communicated to healthcare providers and patients who were responsible for which care process. As a result, patient flows differed locally depending on which institution communicated its functions. Responsibilities were often confused, especially between general practitioners (GPs), emergency physicians, and clinics. There was also a lack of information about patients transferred between, for example, general practice and a clinic, which complicated and multiplied care processes, especially for infectious patients.

“*Because the coordination did not take place as one would have wished. You had the feeling that everyone was blaming the other. No one is responsible for the problem, and the other person has to solve it, the inpatient sector or outpatient sector*.” (clinic 1, female)

When a facility, such as a general practice or a swabbing point for isolated SARS-CoV-2 testing, had to close, for example, due to holidays or illness, participants described that there was no alternative and patients were unable to find care. Lack of resources was also described in terms of safety equipment, staff, information in general, intensive care unit beds, and laboratory capacity.

Social brokers were identified in the role of coordinators and knowledge brokers, for example, in the transformative elements of a nursing home coordination center and a clinic corona phone for information exchange with the outpatient sector.

“*In our case, the university hospital has taken over the coordination. The department of general practice, which then actually always brings all the family doctors and all the established doctors per nursing home under one hat, so to speak*.” (district offices/health authorities 7, male)

In addition, the following individuals and institutions acted as social brokers, which varied from place to place: representatives of the Association of Statutory Health Insurance Physicians, including the so-called “pandemic coordinators,” of the local medical profession, of the local hospital, and of the district offices. In the first two groups mentioned, GPs were also represented, and overall, almost all institutions involved in the primary healthcare system were represented.

These multiple institutions and health professionals communicate with each other individually and in a variety of ways, including (online) meetings, emails, WhatsApp messages, phone calls, letters, and others. Steering committees were formed and implemented individually. They usually included the primary healthcare institutions in different scopes, but also local institutions outside the healthcare system like the local council or civil protection. However, many participants described that sometimes responsible members, like local GPs, were not included in the meetings and individuals did not attend the meetings, resulting in a lack of information transfer. The transfer of knowledge to the public or the involvement of the public in these interfaces played a minor role in the focus groups and interviews. It was mentioned by one GP in the context of requesting test results, but especially by nursing homes, which described active communication with residents and relatives about health status, current regulations, and finding alternatives for visits during lockdowns. One GP stated that she became “*locally famous*” (medical association 4, female, GP) and that the public and the press approached her to ask for her opinion and specific information.

In summary, many different actors were involved in healthcare processes and communication interfaces. There was a lack of coordination and explicit responsibilities among these institutions, although social brokers were identified. There was no clear picture of responsibilities and all the different forms of knowledge and information flow.

These forms of knowledge and information were primarily associated with adaptive and transformative capacity. Healthcare professionals, especially GPs and hospital staff, had to provide regular healthcare as well as new processes due to the pandemic, with the same or fewer staff and resources available. Transformative capacity was primarily mentioned and included by all stakeholders and GPs, for example, the funding of new COVID contact points like swab centers, general practices that specialized in COVID-19 care, external outpatient infection centers, or coordination units.

### 3.2 The ability to anticipate and cope with uncertainties and unplanned events

The analysis of the ability to anticipate and cope with uncertainties revealed heterogeneous characteristics at the individual and system levels in primary care.

At the individual level, heterogeneous behavior was described, especially among physicians. Some were described as retiring and were no longer able to provide healthcare as usual for various reasons that were not clear. For many patients and institutions, such as nursing homes, it was described as difficult to find physicians to provide and cover healthcare, especially during holidays. In addition, some physicians were said to have acted only when financial incentives were offered.

“*Some GPs are really clever, (...) but there are others who are so sluggish that you get the impression they haven't really noticed the pandemic at all. So the differences are substantial, but things are improving*.” (clinic 2, male)

Other physicians were described as highly motivated and saw themselves as responsible for covering care processes and developing coping strategies. These individuals were said to have saved healthcare provision during the first waves and chaotic phases.

“*So, with those you fought outside, that was a real battle, that was outstanding commitment in building the extraordinary structures. There were the GPs in front, the specialists also a few. Even dentists stepped forward. But it was mainly female general practitioners who were carrying the load*.” (medical association 3, male)

Along with these heterogeneous characteristics, physicians were described as gathering and sharing information in different ways. Some were described as trying to keep up with ever-changing regulations and information, while others were described as being slower, less well-informed, and therefore adequately not prepared to fulfill current care processes.

At the system level, the response to the first waves of the pandemic was described as “*chaotic*” and unstructured “*because there was simply no blueprint for this type of crisis and pandemic*” (district offices/health authorities 1, male). Some institutions, such as the ASHIP, were described as responding slowly, and participants felt left alone. Access to consistent information and certain resources, such as security materials and personnel, was described as lacking.

In general, the health system and its response were observed to be slower and more chaotic, except for the aforementioned quick and flexible responses of highly motivated individuals.

Aspects of the ability to respond to uncertainties were assigned mainly to the level of adaptive capacity as it was described, how institutions and individuals reacted to the shock of the pandemic, and how they coped with the associated additional workload. Transformative aspects included responses related to the establishment of new structures, such as taking on the responsibility of acting as a social broker, providing sole care in nursing homes, and participating in the development of new processes and structures.

### 3.3 Interdependence: the capacity to engage effectively with and handle multiple and cross-scale dynamics

Interdependence was mainly observed within the health system. Aspects outside the health system were not described in the focus groups and interviews.

Collaboration, communication, and coordination of care were observed to be crucial. Quality of care and efficiency were observed to be based on good or poor communication between the actors. Some participants described how their close and long-term relationship led to good and efficient communication, for example, between clinics and GP practices, during the pandemic shock.

“*where you had good connections, you could turn to or if you knew someone with whom you had already had a good working relationship before, then that worked to some extent, otherwise not at all*.” (clinic 4, female)

In addition, some described rapid communication by telephone or when the institutions were close together, such as emergency departments and primary care urgent care clinics within a clinic. Communication was also described as “*improved during the pandemic*” (clinic 1, female) due to the need for intensive cooperation and collaboration.

Good communication was described as associated with more efficient healthcare and better quality of care. Care providers were said to follow guidelines, and the work of others was valued. This was observed especially within larger networks with many different actors, but at the local level and when all actors were equally involved. Working in networks was said to have “*benefited us immensely*” (clinic 5, male).

However, as described in Section 3.2, GPs in particular withdrew from their responsibilities, and hospitals or nursing homes took over. On the one hand, this can be seen as a flexible response on the part of the healthcare system, but on the other hand, the actors now in charge expressed frustration and a high workload.

Only a few described feeling a euphoric group dynamic and thinking, “*yes, we can do it*” (medical association 1, female, GP).

In addition to good communication between individuals at the local level, many conflicts were observed as well. If one part did not fulfill its goals, the other part had to take over and sometimes force them to act. Communication was described as poor when actors did not know each other, when some actors were excluded from meetings or excluded themselves, or when healthcare providers did not seem to know each other's responsibilities and workloads.

This results from the lack of knowledge transfer described in Section 3.1. The consequences of this lack of communication and collaboration included, for example, an overload of emergency rooms.

“*We don't know where to put those people, the emergency room is bursting at the seams, regularly. (…) At that point, hardly any GP had said: no, we can't still send everyone to the emergency room, they have enough to do*.” (clinic 6, male)

In general, the relationship between the different healthcare providers was described by some as conflictual. One participant described his impression that “*GPs did not see themselves as part of the system*” and “*it is hard to integrate them into a structure because they are individual entrepreneurs*.” (district offices/health authorities 2, female).

Conflicts between physicians were also described. The biggest conflict is competition between GPs, which inhibits adequate collaboration. GPs were described to have had the “*fear of sending patients to the outpatient infection centres, because they could lose these patients to a colleague*” (clinic 8, male), and as a consequence, they were sent to the emergency room instead.

Social brokers have been described as improving communication and the allocation of responsibilities. Communication with social brokers was described as close. However, the abovementioned conflicts were described despite the presence of social brokers, and communication with social brokers was described as difficult when the respective person was not aware of the situation and the responsibilities of the other actors or did not pass on sufficient information.

The aforementioned aspects of interdependence were assigned to levels of adaptive and transformative capacity. Adaptive capacity included communication between actors that already existed and had to be intensified during the pandemic; transformative capacity included new relationships and communication channels in the context of steering committees, the implementation of social brokers, and new responsibilities that were taken on by other care providers.

### 3.4 Legitimacy: the capacity to develop socially and contextually accepted institutions and norms

Overall, person-centeredness, either regarding patients or healthcare providers, played a minor role within the transcripts. Aspects of patient-centeredness were almost only mentioned by members of care homes, who explained how they coordinated visits, informed patients and relatives, and deliberated with relatives about how contact with patients might be possible and what health preferences their patients had. Patient-centeredness seemed to play a major role in their work, and within the focus group, statements concerning patients' or relatives' preferences, knowledge, or abilities could be observed. In this regard, nursing homes partly took over tasks that GPs were usually responsible for.

“*(…) if someone really had to go to hospital, in many cases it didn't make sense, but in some cases, it was very important to the relatives, even if the general practitioners said: it doesn't make any sense*.” (nursing homes 2, male)

Other aspects of patients' or the public's trust in health institutions became apparent regarding the usage behavior of those institutions. Patients were observed to go into hospitals as their favorite point of contact, whether it was objectively an emergency or not. Many hospitals took over care processes from GPs, which was well-accepted by the public.

“*at the beginning nobody really knew, then one directs to the hospital preferably*.” (clinic 1, female)

Person-centeredness also concerns healthcare providers, for example, regarding the development of care processes, working material, or others. Especially the Association of Statutory Health Insurance Physicians was mentioned on this behalf. Processes were mostly perceived as top-down and had a slow response. Some participants said they and GPs felt “*let down*” (clinic 2, male).

Within the focus group, including members of the Association of Statutory Health Insurance Physicians, experiences concerning cooperation were described as positive and person-centered but also self-critical and too slow because they were dependent on others, such as political decisions. This was observed as a relocation of responsibilities and accusing, which was also partly observed within the other healthcare providers. Especially GPs, hospitals, and the Association of Statutory Health Insurance Physicians were blamed by the respective others for not fulfilling their tasks and for deficient cooperation and communication. Lack of digitization and high bureaucracy were named as having worsened this aspect.

As social brokers, pandemic commissioners and coordination units were primarily named as trusted services associated with positive outcomes and communication. This was mostly mentioned whenever the social brokers were informed about local working procedures, for example, when they were usually working in primary care themselves.

“*I would describe the pandemic commissioner as a milestone. Because he was then widely acknowledged by colleagues*.” (district offices/health authorities 2, female)

Negative and positive perceptions of cooperation and communication were within the data. However, relocating blame and naming problems regarding communication and cooperation occurred despite the existence of multiple different social brokers and occurred in every focus group and interview.

Codes within the dimension of legitimacy could not be easily allocated to the other levels of resilience. Some codes were associated with transformative capacity, for example, concerning social brokers, and some with adaptive capacity, like managing patient visits.

## 4 Discussion

The aim of this study was to explore the resilience of the German primary healthcare system from the perspective of identified interface stakeholders during the second wave of the SARS-CoV-2 pandemic.

### 4.1 Summary of main findings

The main findings include a gap in knowledge transfer on many levels, a resulting unstructured and non-transparent allocation of responsibilities, and unregulated patient flow. GPs and other actors in the different institutions took over the role of social brokers and supported care coordination and knowledge transfer. New, transformative institutions created in response to the pandemic emerged within primary care. The capacity to cope with uncertainties showed a slow and chaotic response at the system level and a heterogeneous response at the individual level. Some GPs showed a fast and proactive response, while others withdrew from their tasks and refused to provide care. Communication was described as good when there was a previous relationship to build on or a familiar point of contact to refer to. In this context, healthcare was described as more efficient and coordinated. Communication between different healthcare providers improved during the pandemic, and networks were described as helpful. Competition between GPs was mentioned as a major barrier to resilience, as well as withdrawing GPs. These conflicts showed the effects of additional workload for the other stakeholders. The function of social brokers and steering committees was socially accepted and described as beneficial. Accusations and blaming others were found in all focus groups and interviews and were perceived as a barrier to resilience.

### 4.2 Interpretation of main findings

According to Blanchet et al. (16), public health outbreaks need a functional disease surveillance system. For managing COVID-19, disease surveillance and the information of healthcare providers therefore play a role in managing the resilience of the German primary care system. However, within this data, disease surveillance played only a minor role, and participants described a lack of knowledge transfer either between the health system and the public or between healthcare providers and institutions. This also led to conflicts concerning the interdependence of healthcare providers. A structure and guidelines for managing responsibilities and tasks were missing, which resulted in chaos and thus reduced the resilience of a health system (16).

GP individuals showed different types of reactions as a response to uncertainties. Some were passive and restrained, and some were active and willing to manage the crisis. These different types were also observed by Stengel et al. (20), who found out the motives behind this behavior, for example, worries about organizational burden and a lack of safety equipment. In this study, individual performance was on the one hand managing the crisis and initiating new care processes and structures; on the other hand, regional differences occurred and healthcare providers felt let down. To discuss the centralized vs. decentralized organization of the health system, France showed a centralized organization that was asked to be revised and decentralized after the first year of the pandemic (30). During the first year, processes and decisions made by the government seemed largely similar to German coping strategies (30). The German National Health Ministry aims for a mix of centralized and decentralized elements, for example, through public health services (31). Within this plan, the national health ministry also aims to improve knowledge transfer to the public and describes health system response as slow (31). Based on the observed regional and interindividual differences in this study, the results can contribute to the development of national resilience strategies.

Blanchet et al. (16) furthermore describe low interdependence on constructs across scales outside of the health system increases resilience, which was observed in this data. However, the resilience framework was developed primarily for analysis at the health system level (16). In the context of this analysis, which focuses on primary care, it would have been more appropriate to focus on this and the health system level as “influences across scale.” Even though social brokers were implemented, socially accepted, and described as having helped coordinate care, major conflicts, and communication gaps were described, reducing the resilience of the health system and resulting in unstructured care flows and inefficiency.

The domain of legitimacy played a minor role in this study and was focused on statements to the subcode “community trust and ownership.” However, person-centeredness is described as important to gain acceptance by institutions and processes and thus may increase user adherence (16). This was not observed in this data. Other research, however, showed increased aggressiveness and dissatisfaction among patients (32). Hence, patient adherence and satisfaction of healthcare providers with guidelines provided to them could have been improved with a higher degree of person-centeredness instead of top-down processes.

In this study, most codes were assigned to transformative capacity, which is observed to be present when a major shock resulting in the highest stress and major structural changes occurs (16). Biddle et al. (15) reported that most empirical studies focused on absorptive and adaptive capacities, transformative capacity, and the domain of legitimacy were underrepresented. As the review of Biddle et al. (15) was done before the COVID-19 pandemic, these aspects may have become more present since then, as the pandemic is perceived as a major shock (3).

Analyzing the level of service delivery and in the context of health services research, this framework showed several limitations. Saulnier et al. (19) described a gap between theory and practice and pledged to a revision of this framework. Furthermore, other, newer resilience frameworks also focus on the health system level (33–35). Haldane et al. (21) developed a resilience framework at the system level that largely meets the results of this study but was developed specifically for resilience in managing COVID-19. On top, the framework by Blanchet et al. (16) focuses on acute coping with shock or stress and does not consider the aspect of recovery and “jumping back,” which is the origin of the term resilience (15) and was part of the gathered data. As a conclusion, a general resilience framework on care level and adapted for use in health services research is indicated, which should merge organizational resilience and individual and workforce resilience in the context of the surrounding health system resilience. In this manner, it could be useful to bring the different resilience frameworks at the health system level together, as the transfer of theoretical foundations and empirical research on resilience is not consistent yet (15).

### 4.3 Strengths and limitations

Due to recruiting issues during the second wave of the pandemic, it was not possible to include participants in all regions of Baden-Wurttemberg, as planned. As local differences could be observed in care processes and structures, the generalization of results is limited. Furthermore, selection bias cannot be excluded, as it can be assumed that primarily highly motivated people followed our study invitation. However, it can be seen as a major strength to have collected and analyzed data during this second wave of the pandemic and be able to paint a precise picture without risking recall bias and an overestimation of positive effects (36).

Within the participants, there were some GPs in a dual role, working in stakeholder institutions, which might support socially accepted answers. However, including GPs in this dual role is seen as a strength as well, as it is, therefore, possible to get a more holistic view of the primary care system.

This study takes a theoretical resilience framework and applies it to the level of service delivery. It is therefore, together with Stengel et al. (20), one of the first studies to specifically investigate the organizational resilience of primary care. Previous studies have focused primarily on specific parts of organizational resilience like lessons learned (37–40) or individual resilience and mental health (23, 41, 42), as well as resilience at the health system level (15, 21, 35). As a conclusion, implications for further research and for strengthening the resilience of primary care can be derived based on this study. To achieve the highest possible validity, additional qualitative aspects such as semi-structured interviews and focus groups on selected topics should be added to further research in order to deepen the key issues in each category. Where appropriate, the integration of quantitative concepts should be considered.

## Data availability statement

The datasets presented in this article are not readily available because as required by the European Data Protection law the participants were informed of the privacy policy and agreed that the pseudonymized data would only be available to the project team and would be stored at the study center. Requests to access the datasets should be directed to Sandra Stengel (sandra.stengel@med.uni-heidelberg.de).

## Ethics statement

The studies involving humans were approved by Ethics Committee of the Medical Faculty Heidelberg. The studies were conducted in accordance with the local legislation and institutional requirements. The participants provided their written informed consent to participate in this study. Ethics committee number: S-418/2020; Approved 2020-06-15.

## Author contributions

NL: Formal analysis, Validation, Visualization, Writing—original draft, Writing—review & editing. SW: Data collection, Data curation, Formal analysis, Investigation, Writing—review & editing. AB: Conceptualization, Project administration, Supervision, Writing—review & editing. CR: Conceptualization, Methodology, Supervision, Writing—review & editing. FP-K: Conceptualization, Supervision, Writing—review & editing. MW: Conceptualization, Funding acquisition, Methodology, Supervision, Writing—review & editing. SS: Conceptualization, Data collection, Formal analysis, Funding acquisition, Investigation, Methodology, Project administration, Supervision, Validation, Writing—original draft, Writing—review & editing.

## Footnotes

^1^Citation format: professional background, pseudonymized number, gender. Specific professions are reported if relevant.
