# Supplementary material for: Resilience of the primary healthcare system: perspectives of German stakeholders at primary care interfaces during the second wave of the COVID-19 pandemic
Source: Front Med (Lausanne). 2024 Apr 24;11:1322765. doi: 10.3389/fmed.2024.1322765 (PMC11076821; doi:10.3389/fmed.2024.1322765)
Supplement: Supplementary file 2 [file Data_Sheet_2.PDF]

Supplementary Table 1 - Overview of all codes

main dimension	subcode	subcode level 2	number of codes
knowledge			total codes = 491
	knowledge about the disease (inductive)		8
	disease surveillance		4
	health status of population and their health priorities		7
	weaknesses in health system		41
	resources	understanding / overview	32
		identification of gaps	45
	knowledge about cooperation / coordination / interfaces	general	44
		communication devices	63
		interfaces	106
		interface to public	22
		identification of social brokers	56
	absorptive capacity		0
	adaptive capacity		15
	transformative capacity		48
uncertainties			total codes = 312
	individual level	general	3
		individual adaptability	62
		individual response to stress / shock	79
		initiative in the development of processes (inductive)	7
		rapid and appropriate decision making	37
	system level	general	41
		access to flexible, adaptable resources	20
		rapid acting, rapid access to resources	32
	absorptive capacity		0
	adaptive capacity		13
	transformative capacity		9

interdependence			total codes = 392
	social brokers	general	5
		positive interdependence and resulting effects	38
		conflicts and resulting effects	18
	across scales / health care providers	general	14
		positive interdependence and resulting effects	174
		conflicts and resulting effects	122
	absorptive capacity		0
	adaptive capacity		14
	transformative capacity		7
legitimacy			total codes = 133
	community trust and ownership	general	6
		concerning interfaces / health care providers / institutions	60
		concerning social brokers	20
		resulting effects	8
	person centredness	patients	24
		health care providers	9
	client based information system on quality of care		0
	absorptive capacity		0
	adaptive capacity		0
	transformative capacity		6
